# Potential of Mitochondrial Genome Editing for Human Fertility Health

**DOI:** 10.3389/fgene.2021.673951

**Published:** 2021-07-20

**Authors:** Lin Fu, Yu-Xin Luo, Ying Liu, Hui Liu, Hong-zhen Li, Yang Yu

**Affiliations:** ^1^Beijing Key Laboratory of Reproductive Endocrinology and Assisted Reproductive Technology and Key Laboratory of Assisted Reproduction, Ministry of Education, Center of Reproductive Medicine, Department of Obstetrics and Gynecology, Peking University Third Hospital, Beijing, China; ^2^Key Laboratory for Stem Cells and Tissue Engineering, Ministry of Education, Sun Yat-sen University, Guangzhou, China; ^3^Food Inspection and Quarantine Technology Center of Shenzhen Customs District, FICS, Shenzhen, China; ^4^Stem Cell Research Center, Peking University Third Hospital, Beijing, China

**Keywords:** mitochondrial DNA, mitochondria DNA mutation, mitochondrial genome editing, human infertility, gene therapy

## Abstract

Mitochondrial DNA (mtDNA) encodes vital proteins and RNAs for the normal functioning of the mitochondria. Mutations in mtDNA leading to mitochondrial dysfunction are relevant to a large spectrum of diseases, including fertility disorders. Since mtDNA undergoes rather complex processes during gametogenesis and fertilization, clarification of the changes and functions of mtDNA and its essential impact on gamete quality and fertility during this process is of great significance. Thanks to the emergence and rapid development of gene editing technology, breakthroughs have been made in mitochondrial genome editing (MGE), offering great potential for the treatment of mtDNA-related diseases. In this review, we summarize the features of mitochondria and their unique genome, emphasizing their inheritance patterns; illustrate the role of mtDNA in gametogenesis and fertilization; and discuss potential therapies based on MGE as well as the outlook in this field.

## Background

### Introduction of the Mitochondria and mtDNA

Mitochondria are double-membrane organelles producing more than 90% of adenosine triphosphate (ATP) through oxidative phosphorylation (OXPHOS). Additionally, mitochondria play a part in essential biological processes, including biosynthesis, calcium storage, cellular signaling, and immune response (Dyall et al., 2004; Nakahira et al., 2011; Collins et al., 2012). The electrochemical disequilibrium potential across the inner mitochondrial membrane (IMM) that is generated through proton pumping by respiratory chain complexes I, III, and IV enables the facilitation of the intricate functions above (Nunnari and Suomalainen, 2012). As the only organelle that has its own genetic information stored spatially and heritably separate from the nucleus, mitochondria are regulated by both the mitochondrial genome and the nuclear genome (Nass and Nass, 1963; Riepsaame, 2020). However, although more than 1,000 mitochondrial proteins have been identified or predicted, making up ~ 10% of the total cellular proteome, most of them are encoded by nuclear genes (Pagliarini et al., 2008). Hence, it could be complicated to determine the respective and reciprocal effects of the two sets of genomes.

In contrast to the nuclear DNA (nDNA), the mitochondrial DNA (mtDNA) is a double-stranded circular molecule [an outer heavy strand (H-strand) and an inner light strand (L-strand)] containing 16,569 base pairs and 37 genes (Anderson et al., 1981). Additionally, there are two noncoding regions of mtDNA: One is the origin of replication of the light strand (OL), and the other is the displacement loop (D-loop), also named the control region (CR), which includes the origin of replication of the heavy strand (OH), the heavy-strand promoter, and the light-strand promoter (Lott et al., 2013). The mtDNA of mammalian cells is relatively small and genetically compact, containing two overlapping genes and very little noncoding sequence (Ojala et al., 1981). The mtDNA replicative mechanism is somewhat controversial, and recent studies support the originally proposed strand-displacement mechanism. Robberson and Clayton put forward the strand-displacement model in the 1970s, according to which the mtDNA synthesis initiates from two distinct replication origins (the origin of heavy-strand replication, OriH and the origin of light-strand replication, and OriL) and proceeds to opposite directions (Robberson and Clayton, 1972). In 2000, Holt et al. proposed the strand-coupled model, which suggests a coupled and bi-directionally replication of the heavy- and light-strand starting from an initiation zone downstream of OriH (Holt et al., 2000). Later studies add modification to this theory, suggesting the existence of extensive RNA priming of the lagging strand, which is subsequently replaced by DNA (Yang et al., 2002; Yasukawa et al., 2006). According to the RITOLS (RNA incorporation throughout the lagging strand) model, RNA intermediates initiate lagging strand DNA synthesis discontinuously *via* several small RNA primers (Yang et al., 2002; Yasukawa et al., 2006; Phillips et al., 2017). In addition to the mitochondrion being a semiautonomous organelle, mitochondrial inheritance includes other interesting features: (1) The polyplasmy of mtDNA. Unlike nDNA, a single cell in the human body usually contains hundreds of mitochondria, each of which harbors 2–10 copies of the mtDNA (except the platelet and unfertilized oocyte). If all the mtDNA molecules in a single cell or tissue are the same, it is called homoplasmy; otherwise, in the case of the mtDNA mutation, we have heteroplasmy (Clay Montier et al., 2009, Wallace and Chalkia, 2013). (2) Differences in the codons. Several codons of mitochondrial inheritance have distinct meanings from those in the nuclear system (commonly AUA, UGA, AGA, and AGG; Gorman et al., 2015). Additionally, the compatibility of tRNA is much higher in the mitochondria, in which 22 tRNAs recognize all the codons of mRNA. (3) Maternal inheritance. The pattern of mitochondrial genome inheritance is uniparental, specifically maternal in mammals (Al Rawi et al., 2011, Zhou et al., 2016). There are 250,000 mtDNAs for each oocyte in humans, but only a small proportion (2~200) can enter the mature oocyte and be passed on to children. This dramatic decrease in mtDNA during oogenesis is named the “bottleneck” effect, which restricts the number of mutant mtDNAs passed down to the offspring (Cree et al., 2008; Gorman et al., 2015; Moraes, 2019). (4) Replicative segregation. Mutant mtDNAs and wild-type mtDNAs are randomly allocated during both mitosis and meiosis, resulting in variations in the proportion and type (Yang et al., 2002; Yasukawa et al., 2006; Phillips et al., 2017) of mtDNAs in the daughter cells (Bogenhagen and Clayton, 1977; Wallace and Chalkia, 2013). Meanwhile, the proportion of mutant to wild-type mtDNA will drift to pure wild-type genes over time (Stewart et al., 2008; Stewart and Chinnery, 2015). (5) High mutation rate (Khrapko et al., 1997). The rate of sequence evolution is 10~20 times higher in mtDNA than in nDNA (Brown et al., 1982; Wallace et al., 1987). Due to the high mutation rate, mtDNA is extremely polyplasmic, especially the D-loop region, which contains two areas characterized by high polyplasmy [hypervariable regions I and II (HVI and HVII)].

### Mitochondria and mtDNA in Human Fertilization

During gametogenesis, mitochondria undergo a very dynamic process of quantitative structural and compositional change, usually from an orthodox pattern into a denser and more activated pattern with the matrix condensed and the cristae compacted (Motta et al., 2000; Ramalho-Santos et al., 2009). In the process of spermiogenesis, a large proportion of the cell cytoplasm (including mitochondria) is eliminated in the form of residual bodies in the spermatogonia and early spermatocytes, and only a few mitochondria persist in the mature spermatozoa, with their shape elongated (DeLuca and O’Farrell, 2012; Park and Pang, 2021). Because of this elimination combined with the downregulation of mitochondrial transcription factor A (TFAM) protein levels, a drastic decrease in the mtDNA copy number during spermatogenesis occurs (Rantanen et al., 2001). On the other hand, during oogenesis, the mitochondria tend to be oval and have a dense matrix with few cristae; central clumping around the pronuclei is also observed (Sathananthan and Trounson, 2000; Motta et al., 2003). Moreover, following the “bottleneck” effect, where primordial germ cells lose a large proportion of their mtDNA, the number of mitochondria is globally amplified, producing tens of thousands of copies of mtDNA and making mature oocytes the most mitochondria-dense cells in any organism (average approximately 250,000 copies in humans; Otten and Smeets, 2015; Floros et al., 2018; Zhang et al., 2018). During early embryogenesis, active destruction and elimination of the paternal mitochondria and mtDNA occur, causing the mitochondrial genome to be exclusively maternally inherited from the oocyte (Sato and Sato, 2013; Carelli, 2015).

Structural changes result in highly active oxidative metabolism and ATP production as well as modification of the supply of energy substrates. Evidence has shown that mitochondrial metabolism may not be required during oogenesis; as a result, the oocyte mitochondrial network is fragmented, and each contains a small number of mtDNA molecules, supporting an immature state and lower mitochondrial activity (Motta et al., 2000, 2003). Moreover, oocyte energy production relies essentially on pyruvate as a substrate, leading to ATP generation through OXPHOS within the electron transport chain (ETC), since glycolysis in oocytes is restricted by low phosphofructokinase expression (Collado-Fernandez et al., 2012; Dalton et al., 2014). Unlike oocytes, cumulus cells have good glycolytic capacity; they confer metabolic support to oocytes by providing pyruvate *via* gap junctions (Dalton et al., 2014; Clarke, 2018). Consequently, it is well accepted that granulosa/cumulus cells supply most of the energetic molecules required by the oocyte during early folliculogenesis (Collado-Fernandez et al., 2012). Nonetheless, with cumulus cell expansion, mitochondria reassume a prominent role during oocyte maturation (Muller et al., 2004). In this way, mitochondria tend to distribute and localize in ooplasmic areas with relatively high ATP demand for energy-consuming events leading to oocyte cytoplasmic and nuclear maturation, including germinal vesicle breakdown and microtubule assembly and disassembly for meiotic spindle formation (Yu et al., 2010; Coticchio et al., 2015). In spermatogenesis, energy substrate resources are converted from the direct blood nutrient supply to lactate and pyruvate provided by Sertoli cells along with the transport of male germ cells across the germinal epithelium from the basal compartment to the central compartment (O’Shaughnessy, 2014). To meet the energetic demand of flagellar movement and the subsequent fertilization processes, it has been proposed that ATP is produced by mitochondria through OXPHOS in the midpiece and that anaerobic glycolysis provides ATP in the principal piece in spermatozoa (Gu et al., 2019). In addition to the mutually transformational energy sources pyruvate and lactate, it has been shown that fatty acids could also supply energy *via* β-oxidation within mitochondria and provide energy for spermatogenesis, sperm maturation, and flagellar movement in humans (Ferramosca et al., 2008). Since the energy supply is important to the normal functioning of gametes, any disturbance or mutation occurring in the mitochondria could affect germ cell quality and further influence the fertility of the organism. And understanding the role of mtDNA in human infertility is the premise of exploring novel treatments of the currently intractable infertility problems.

### Mitochondria Genome Editing

With the development of gene editing technology, more specific and targeted methods that can generate nonnative mtDNA sequences or repair the sequences of existing mtDNA to shift the mtDNA heteroplasmy ratio have become possible. Consequently, mitochondrial genome editings (MGEs) have become a novel hotspot for researchers. Additionally, the centrality of mitochondrial gene function and the threshold effect of mitochondrial diseases mean that any genetic therapy must probably correct the majority of the cells in the body to make germline correction likely (Roth and Marson, 2021). It was first reported in 2015, that specific reduction of mitochondrial genomes in the germline for preventing transmission of mitochondrial diseases could be achieved through mitochondria-targeted nucleases (Reddy et al., 2015). Mitochondrial genome editing refers to modification of human zygotes or oocytes at risk for mtDNA disease *via* the intracytoplasmic microinjection of mitochondria-targeted nucleases in order to preclude the germline transmission of mutant mtDNA haplotypes. Targetable nucleases, such as endonucleases, zinc finger nucleases (ZFN), or transcription activator-like effector nuclease (TALEN), clustered regularly interspaced short palindromic repeats (CRISPR) and CRISPR-associated protein 9 (Cas9)—CRISPR/Cas9 could enable direct and precise gene editing of the mitochondrial genome. As an embryo-sparing donor independent method for mtDNA diseases prevention, MGE harbors significant potential as an option for mitochondria-related infertility treatment.

Mitochondria-targeted restriction endonucleases (mitoREs) are endonucleases recombined with the N-terminus polypeptides—mitochondrial localization signals (MLS) with the help of which the protein could be directed into the mitochondria. Expressing in the nuclear–cytosolic compartments, the mitoREs recognize specific sequences in the mtDNA and cause double-strand breaks (DSBs) once imported into the mitochondria (Srivastava and Moraes, 2001). Initially developed for the nuclear genome editing, the ZFNs and TALENs consist of the MLS, the DNA-binding domain, and the nuclease catalytic domain fused in one protein (Bacman et al., 2013; Gammage et al., 2014). Double-strand breaks are produced *via* a nuclease domain taken from FokI nuclease (Sugisaki and Kanazawa, 1981). And the zinc finger DNA-binding domain or the bacterial TALE protein is used to promote the binding specificity of engineered nucleases with pathogenic mtDNA molecules (Bacman et al., 2013, Gammage et al., 2014). As a novel nuclear genome editing technology, CRISPR/Cas9 employs a single-guide RNA (sgRNA) to edit the DNA target of specific sequences (Jinek et al., 2012). Since the DNA recognition is based on DNA–RNA binding, it is more specific compared to other methods. Moreover, it is believed to be more effective in genome editing and more flexible due to a simple procedure of the sgRNA customization (Hsu et al., 2014).

Manipulation of mtDNA *via* MGE could be further divided into the perturbing of mtDNA copy number as well as the repair and elimination of pathogenic mutant mtDNA (Prole et al., 2020). On the one hand, cleavage and breakdown of mtDNA has been achieved using mitochondria-targeted TALENs (mito-TALENs; Bacman et al., 2013, Yang et al., 2018). and mitochondria-targeted ZFNs (mito-ZFNs; Gammage et al., 2014, 2016), which could reduce the mutant mitochondrial genome. On the other, the potentially pathogenic mutant mtDNA can be edited to reduce the mtDNA heteroplasmy into the normal wild type using mito-REs (Srivastava and Moraes, 2001), mito-TALENs (Gammage et al., 2018a,b), or mito-ZFNs (Pereira et al., 2018).

## mtDNAs in Human Infertility

Mitochondrial diseases are defined by a group of genetic disorders caused by mutations in the mitochondrial genome and nuclear genomes that encode proteins involved in mitochondrial function, resulting in defects in OXPHOS (Gorman et al., 2016). Mitochondrial DNA disorders can lead to widespread symptoms and commonly affect organs with higher energy requirements, causing cardiovascular, neurological, and age-related degenerative diseases (Kauppila et al., 2016). Additionally, both harmful environmental factors and pathogenic mutations in mtDNA could lead to the occurrence of disease. Nevertheless, due to the protective and compensatory effects of wild-type mtDNA, mutations in mtDNA do not lead to severe consequences immediately (Wallace and Chalkia, 2013). The disease severity depends on the proportion of a particular mutation; only when the mutant mtDNA percentage reaches a certain biochemical threshold (usually > 70–90%) will the phenotype become clinically visible (Morganhughes et al., 1995; Mustafa et al., 2020).

**Figure 1:**
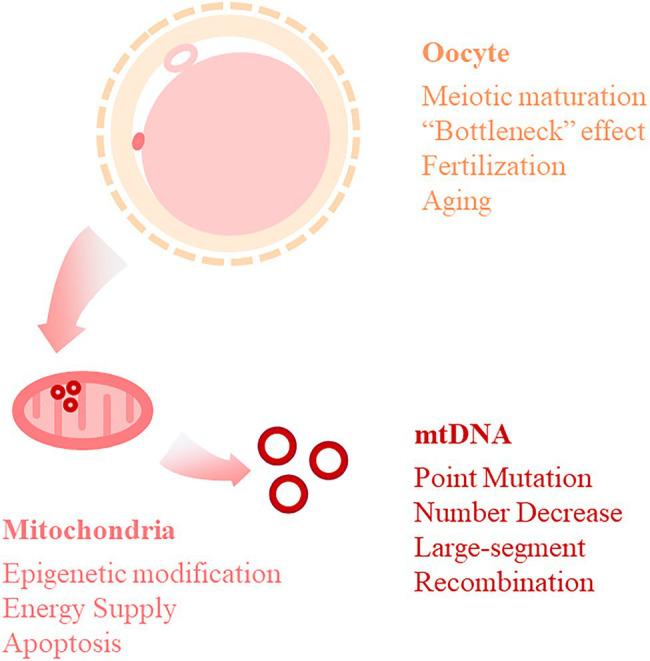
Summary of mitochondria and mtDNA function in oocyte.

For the human reproductive system, mitochondria and mtDNA are receiving increasing attention for their role in the genesis of gametes and moreover in the pathogenic fertilization process. Here, we summarize the impact of mitochondria and mtDNA on male and female infertility based on the developmental stages of germ cells. (Figure 1 summarized the function of mitochondria and mtDNA in spermatozoa and oocyte as well as the possible pathogenic process).

### mtDNA in Male Infertility

Generally, male reproductive function seems particularly vulnerable to mitochondrial dysfunction associated with mtDNA abnormalities (Vaught and Dowling, 2018). Defects in sperm ETC functioning have been proven to be associated with sperm quality impairment (Boguenet et al., 2021). In this respect, oxygen consumption and mitochondrial respiration efficiency are correlated with sperm motility (Sousa et al., 2011; Ferramosca et al., 2012). Moreover, studies have shown that the mitochondrial membrane potential, which reflects mitochondrial functioning and energy status, is correlated with acrosome reaction capability in humans as well as sperm fertilizing capability in natural conception and *in vitro* fertilization (IVF; Marchetti et al., 2012, Voncina et al., 2016). Additionally, specific recognition and inhibition of each of the respiratory chain complexes have been shown to impact sperm motility and viability (Ruiz-Pesini et al., 2007). Through genomic and proteomic technology, alterations in the expression of mitochondrial genes encoded by mitochondrial and nuclear genomes (Jodar et al., 2012) as well as modifications in the expression of several mitochondrial proteins have been found in the spermatozoa of asthenospermic patients (Nowicka-Bauer et al., 2018; Moscatelli et al., 2019). Consequently, mitochondria play a key role in sperm physiology and motility, and low sperm motility might have a strong relationship with dysfunctional mitochondria and insufficient energy production (Boguenet et al., 2021).

Point mutations and major rearrangements have been concluded to be the two primary types of mtDNA alterations. First, an increased incidence of certain point variants in mtDNA is represented in oligo and/or asthenospermic patients and patients with low fertility rates (Siwar et al., 2014; Mao et al., 2015; Ni et al., 2017). However, due to the neutrality and uncertainty of the mutation as well as the relatively small scale of the studies, it remains difficult to draw a firm conclusion about the connection between the point mutation and the disease (Vertika et al., 2020; Boguenet et al., 2021). Second, major rearrangements of sperm mtDNA, consisting mainly of deletions, have been shown to be linked to sperm quality. The deletion of 4,977 bp has been frequently reported to be associated with modified sperm motility and sperm count (Namaghi and Vaziri, 2017, Gholinezhad et al., 2019, Wu et al., 2019, Al Zoubi et al., 2020). Other deletions, multiple deletions in particular, have also been shown to be more frequently found in sperm from infertile patients (Boguenet et al., 2021). According to studies on sperm fractions obtained from density gradient analysis, normal spermatozoa are found in high-density fractions containing more wild-type mtDNA (O’Connell et al., 2003).

Since fertility is not the major concern for patients diagnosed with mitochondrial diseases, studies on male infertility in patients with mitochondrial diseases, especially of the sperm, are rather limited. The few studies have focused particularly on patients with the pathogenic mutation m.3243A>G and have shown a decrease in sperm motility (Spiropoulos et al., 2002). A British investigation of the fertility of a cohort of male patients with mitochondrial disease showed a 35% decrease in their fertility compared to the general UK population (Martikainen et al., 2017). Similar results have been shown in transgenic mouse studies (Trifunovic et al., 2004; Nakada et al., 2006). Other studies have focused on blood mitochondria for mtDNA mutations in infertile patients. Mitochondrial DNA is highly polymorphic with respect to some single-nucleotide polymorphisms (SNPs), especially the mtDNA haplogroups that characterize diverse human population groups (Montiel-Sosa et al., 2006; Gomez-Duran et al., 2010; Feng et al., 2013). Studies have suggested that such haplogroups tend to be sub/overrepresented in individuals with asthenospermia among different population groups. Moreover, Lu et al. (2015) showed in a large-scale study that two-specific SNPs already associated with mitochondrial pathologies, m.16179C>T and m.12361A>G, were related to alterations in sperm count and/or sperm motility (Lu et al., 2015). The same team also demonstrated in 2017 that the m.11696G>A mutation was associated with a significantly higher risk of asthenospermia (Lu et al., 2015; Ji et al., 2017).

### mtDNA in Female Infertility

Sufficient energy supply is essential for satisfactory oocyte quality, which enables a positive reproductive result. It has long been believed that mitochondria act as a key determinant of infertility in women based on their major role in oocyte meiotic maturation (Chiaratti et al., 2018). Moreover, with maternal aging becoming an increasingly notable factor restricting normal pregnancy, mitochondrial dysfunction with aging might explain the increased rates of spindle and chromosomal abnormalities in the oocytes of aged women (May-Panloup et al., 2016; Chiang et al., 2020). In support of this, various studies have shown that mitochondria present dysfunction in oocytes with poor quality (Lee et al., 2014; Boucret et al., 2015; Zhang et al., 2016b). Additionally, it has been suggested that caloric restriction and treatments that prevent organelle damage and stimulate mitochondrial function improve oocyte quality, which further limits the impact of aging on fertility (Zhou et al., 2014, Ben-Meir et al., 2015, Cabello et al., 2015). The accumulation of mtDNA mutations with aging might be the reason for this phenomenon. On the one hand, mtDNA could either rearrange in the anoxic environment of the ovary, which limits OXPHOS and the production of potentially deleterious reactive oxygen species (ROS; Van Blerkom, 2011), or become mutated or deleted during the IVF process with an estimated rate of up to 50% (May-Panloup et al., 2016). On the other hand, the purification process of the “bottleneck” effect might not be efficacious enough to remove some heteroplasmic point mutations (Ye et al., 2014; Floros et al., 2018), so that these pathogenic mutations may multiply later in life in a fraction of the cells, leading to age-related mitochondrial dysfunction (May-Panloup et al., 2016). Finally, inherited and acquired mtDNA mutations may function together to aggravate the phenotype of aging and deleterious consequences of infertility (Greaves et al., 2014; Stewart and Chinnery, 2015).

In addition, recent studies have revealed the correlation between mitochondrial dysfunction and other reproductive diseases or biological processes that could lead to female infertility. First, regarding oocyte metabolism, mitochondria are closely related to apoptosis pathways, such as those involving Ca^2+^, cyclic adenosine monophosphate, and ROS (Tiwari et al., 2015; Chiaratti et al., 2018). Recently, Feng et al. showed that mitochondrial activity played a critically important role in the maintenance of intracellular Ca^2+^ ([Ca^2+^]i) concentration oscillations during oocyte activation *via* the Na^+^/Ca^2+^ exchanger (NCLX) and voltage-dependent Ca^2+^ channel (Wang et al., 2020), which proved the vital role of mitochondrial function in human fertility. Second, Liang et al. demonstrated that the accumulation of mtDNA mutations decreased fertility by impairing the NADH/NAD^+^ redox state of oocytes, which could be rescued by nicotinamide mononucleotide treatment (Yang et al., 2020), revealing the controlling metabolic mechanism behind the deleterious effect of age-accumulated mtDNA mutations on fertility. Third, mitochondrial metabolites could affect oocyte quality *via* epigenetic modification (Titus et al., 2015; Matilainen et al., 2017; Chamani and Keefe, 2019). Studies have shown that metabolites produced by mitochondria (acetyl-CoA, α-ketoglutarate, and S-adenosylmethionine) could remodel chromatin and then regulate genetic expression by influencing histone activity (Montgomery et al., 2015; Menzies et al., 2016; Qi et al., 2019), which implies the critical role of mitochondria in modifying epigenetics in human oocytes. Fourth, for patients with infertility-associated diseases, such as endometriosis or polycystic ovary syndrome (PCOS), mitochondrial dysfunction and mutation have been observed (Zeng et al., 2020). A recent study reported the underlying mechanism of the decline in steroidogenesis caused by mitochondria in human granulosa cells, which led to a decreased fertilization rate, oocyte maturation rate, and oocyte quality and ultimately jeopardized fertility (Urs et al., 2020). Additionally, as shown by a recent study in the Chinese population, mtDNA D-loop alterations and haplotype A15 (*p* adjusted < 0.01) appear to confer resistance to PCOS (Deng et al., 2021).

Consequently, it was proposed that mitochondrial function and mtDNA copy number can be used as biomarkers for the prediction of oocyte quality (Boucret et al., 2015; Fragouli and Wells, 2015). Direct detection of the mtDNA content has yielded an expected correlation with oocyte quality because it has been shown to be decreased in oocytes, cumulus cells, and polar bodies from infertile and aged women (Murakoshi et al., 2013; Konstantinidis et al., 2014; Keefe, 2015). Furthermore, in older women, blastomeres biopsied from cleavage-stage embryos also contained decreased levels of mtDNA (Murakoshi et al., 2013; Diez-Juan et al., 2015; Fragouli et al., 2015), supporting and enabling its clinical application in assisted reproduction. It is plausible that replenishing mitochondria to a suitable degree might counteract mitochondrial decline in defective or aging oocytes (Qi et al., 2019). Since mtDNA plays an important role in human fertility, and that the dysfunction of mitochondria is closely related to the infertility problem in both males and females, it is of great significance to target the mitochondria, especially the mtDNA as treatments of infertility.

## Infertility Treatments Targeting mtDNAs

### Current Nonediting Treatments of Infertility Targeting Mitochondria

There are two main strategies for targeting mitochondria to improve fertility: One is to enhance mitochondrial quality, which mainly includes the use of pharmacologic agents capable of protecting against oxidative stress or increasing the overall efficiency of energy production and calorie restriction (Cecchino et al., 2018); the other is cytoplasmic and mitochondrial transfer therapy developed to prevent the transmission of severe hereditary mitochondrial diseases caused by pathogenic mutations of mtDNA (Adashi and Cohen, 2018). The bioactive molecules or antioxidants applied in mitochondria-related fertility enhancement in recent years mainly include coenzyme Q10, resveratrol, and alpha-lipoic acid (Cecchino et al., 2018). However, most antioxidant therapies have been applied only in animal models, and their effects and mechanisms in humans are not fully understood. Moreover, as shown in a recent meta-analysis study, the use of antioxidants failed to present a real beneficial impact in terms of pregnancy and live birth rates, and thus, its efficacy in improving fertility seems quite controversial (Showell et al., 2020). Mitochondrial replacement therapy techniques consist of different protocols depending on the part transfused: polar body transfer, maternal spindle transfer, germinal vesicle transfer, and pronuclear transfer (PNT). Zhang et al. (2016a) reported the first case of PNT in a 30-year-old nulligravida woman who had two failed IVF cycles characterized by all her embryos arresting at the two-cell stage using donor oocytes (Zhang et al., 2016b). While studies have demonstrated gradual loss of donor mtDNA and reversal to the nDNA-matched haplotype in MRT derivatives (Tachibana et al., 2018). Kang et al. have shown that although the majority of human ESC lines containing > 99% mtDNA haplotype from donor in MST, some ESC lines demonstrated gradual loss of donor mtDNA and reversal to the maternal mtDNA haplotype (Kang et al., 2016). It is speculated that these polymorphisms may contribute to a replication bias toward particular mtDNA haplotype, and matching patient and cytoplast donors for mitochondrial haplotypes might be considered while performing MRT in humans. Nevertheless, cytoplasmic and mitochondrial transfer therapy remains debatable, not only because of safety and efficacy reasons but also because of ethical concerns, as cytoplasmic injection was banned by the FDA in 2002. Moreover, an offshoot of this technique, autologous germline mitochondrial energy transfer, which refers to the injection of mitochondria derived from the oogonial precursor cells of the patient for the treatment of infertility, has been authorized in a few countries as a therapeutic option for women with a history of multiple IVF failures (Tilly and Sinclair, 2013). Regardless, the development of these techniques pushes forward the application of mitochondrial transfer in the treatment of several types of infertility, most notably those due to ovarian aging or embryo cleavage arrest (Zhang et al., 2016b). However, before these techniques become widely available, studies are required to assess the short-, middle-, and long-term risks to offspring (Dobler et al., 2018). Although the first meta-analyses on the subject have yielded conflicting results, making the practical efficacy of the above transfer techniques rather unclear (Eyre-Walker, 2017; Dobler et al., 2018; Eyre-Walker, 2019), they open the door to a possible solution for the further use of mtDNA editing technologies to improve fertility.

### Potential Treatments Involving MGE

Zinc finger nucleases are dimers composed of the Cys2His2 zinc-finger DNA-binding domain and the FokI DNA-cleavage domain. The DNA-binding domain can be modified to recognize a relatively long and specific target sequence, and the FokI DNA-cleavage domain will induce DSBs once dimerized. Processes have been developed using mito-ZFNs in patient-derived cybrid cells and mouse models. Transcription activator-like effector nucleases have a similar structure to ZFNs, with two DNA-binding domains, one binding specifically to the mutant mtDNA site and the other to both wild-type and mutant mtDNA (Kim and Kim, 2014). Remarkably, mito-TALENs have also been used to target mitochondrial myopathy, encephalopathy, lactic acidosis, and stroke-like episode (MELAS) mutations in induced pluripotent stem cells (iPSCs; Yahata et al., 2017). A study showed that engineered mito-TALENs targeting the m.13513G> A mutation decreased mutant mtDNA levels after transduction (Yahata et al., 2017). Another report demonstrated that an iPSC with the m.3243A> G mutation was successfully eliminated, both in iPSCs and in porcine oocytes, *via* direct injection of mito-TALEN mRNA, suggesting the possibility of applying this method to prevent the transmission of mutant mtDNA in advanced assisted reproductive techniques (Yang et al., 2018; Zekonyte et al., 2020). Although versatile, the generation of novel nucleases for every genomic target requires time-consuming protein engineering and assembly processes *via* ZFNs and TALENs (Dan et al., 2021).

First demonstrated in 2012 (Jinek et al., 2012), CRISPR/Cas9 genome editing technology has successfully captured public attention for its revolutionary influence on gene editing, gaining it the Nobel Prize in Chemistry in 2020. Cas9 can be easily programmed by a guide RNA (gRNA) to target nearly any gRNA-complementary sequence followed by a protospacer-adjacent motif which has provided solutions to miscellaneous and difficult human genetic diseases. CRISPR-associated protein 9 (Cas9) is flexible and effective in genome editing due to the simple procedure of single-gRNA customization. Currently, there is a report claiming that CRISPR/Cas9 can feasibly be used to cleave mtDNA in HEK-293 T cells by expressing gRNAs specific to the mtDNA genes Cox1 and Cox3 for adequate Cas9 localization to mitochondria, and a reduction of almost 80–90% in the intact mtDNA in HEK-293 T cells was reported (Jo et al., 2015; Gammage et al., 2018a). Nonetheless, the import of RNA into mitochondria is naturally infeasible, making it the main obstacle for future research (Zekonyte et al., 2020). Hence, extensive efforts have been devoted to optimizing this system to eliminate mutant mtDNA and edit the MGE, but the approach is still quite controversial, and the application of this technology to effective MGE is far from unambiguous confirmation.

Nonetheless, Beverly et al. recently created an RNA-free DddA-derived cytosine base editor (DdCBE), which consists of nontoxic split halves of the cytidine-deaminating catalyzer DddA, transcription activator-like effector array proteins, and an uracil glycosylase inhibitor. DddA-derived cytosine base editor is capable of catalyzing C·G-to-T·A conversions in human mtDNA with high target specificity and product purity (Mok et al., 2020). However, its application in the correction of unknown pathologies still requires further improvement and perfection of this technology, since it has to date been restricted by the strong preference for a 5'-TC-3' editing sequence. Although it is currently more like a conception of treating human infertility by means of MGE, with the rapid development of MGE technology, feasible methods are sure to be developed in the near future. (Table 1 summarized comparison of different mitochondrial intervention).

**Table 1:** Comparison of different mitochondrial intervention.

Mitochondrial Intervention	Explanation	Specific	Autologous
Mitochondrial replacement therapy			
PNT	Transfer of the patients’ pronuclei	−	−
PBT	Transfer of the patient’s polar body	−	−
MST	Transfer of the patient’s MII spindle complex	−	−
Mitochondrial gene transfer			
Viral vectors	Adenovirus and its associated vectors	−	−
Nonviral Strategy	Gene overexpression, gene knockdown, and chemical agents	−	+
Mitochondrial gene editing			
Mito-RE	Specifically targets and cuts DNA at RE-binding site	+	+
Mito-CRISPR	Specifically cuts DNA at CRISPR/Cas9-binding site	+	+
Mito-ZFN	Specifically targets and cuts DNA at ZFN-binding site	+	+
Mito-TALEN	Specifically targets and cuts DNA at TALEN-binding site	+	+
DdCBE	MtDNA base editor sequence via fused TALE	+	+
*In vitro* Oogenesis	Oocyte derived from selected patient iPSC lines	+	+
Medication	Drug therapy for the enhancement of mitochondria quality	−	+

## Outlook

### Nonediting Strategies of mtDNA Manipulation

The manipulation of mtDNA within cells can be achieved *via* diverse methods, including chemical methods, enzymatical methods, and the manipulation of regulatory proteins or cellular organelles. Prior to MGE technology, carbonyl cyanide m-chlorophenyl hydrazone, 1-methyl-4-phenyl-1,2,3,6-tetrahydropyridin, and rotenone were widely used to impair the ETC to induce mitophagy or oxidative stress-induced neurodegeneration (Jo et al., 2015). Additionally, restriction enzymes, such as EcoRI, PstI, or XhoI, can be targeted to mitochondria used to decrease or eliminate mtDNA in cells. Manipulation of the mtDNA copy number can also be achieved by alteration of the mtDNA replication machinery (Bacman et al., 2014; Prole et al., 2020). Overexpression or knockdown of the key component TFAM will increase or decrease the mtDNA number (Lewis et al., 2016; Desdin-Mico et al., 2020). Moreover, nucleoside analogs, such as the antiretroviral drug zalcitabine that inhibit mitochondrial POLG and cause a decrease in mtDNA copy number, have been used to manipulate mtDNA counts in cells (Young and Copeland, 2016). Moreover, it is predicted that alterations in mitochondrial network fragmentation, which is intimately involved in mechanisms of mitophagy, including Drp1, Opa1, and Mfn1/2, may also enhance the breakdown of mtDNA (Giacomello et al., 2020; Prole et al., 2020).

### Current Status of MGE

With the rapid development of gene technology, our knowledge of the mitochondrial genome and its pathology as well as potential strategies to cope with pathology has made compelling progress. Although treating infertile patients with MGE technology remain a proof-of-concept to some extent, the technology still shows great promise for further study and research on mitochondrial diseases. However, barriers do exist as: It is particularly challenging to target the mitochondrial genome using gene therapies because of the unique inheritance of mitochondria, the rapid onset of phenotypes after fertilization, the copious numbers of mtDNAs per cell, and the physical and chemical barriers surrounding them (Patananan et al., 2016). Similarly, the compactness of the mitochondrial genes and the double-membrane structure make the nontargeted insertion of new genetic material into the mitochondrial genome impractical (Roth and Marson, 2021).

Targetable nucleases also have their strengths and weaknesses:

Mitochondria-targeted restriction endonucleases (mitoREs) are recombinant restriction endonucleases that are expressed in the nuclear-cytosolic compartments and reach the organelle thanks to MLSs, which are N-terminal polypeptides that help direct the protein into the mitochondria. Once imported, mitoREs recognize specific sequences in mtDNA and cause DSBs. This method has been used to selectively cleave one type of mtDNA in a heteroplasmic cell or mouse line, causing shifts in heteroplasmy. Although the approach has proven effective and nondetrimental at cleaving mutant mtDNA and shifting heteroplasmy in cellular (Bacman et al., 2012) and mouse models (Bacman et al., 2010, 2012), it is limited by the paucity of unique recognition sites created by pathogenic mutations (Zekonyte et al., 2020).

Precise and efficient genome editing using meganucleases, including ZFNs, TALENs, and Cas proteins, has attracted increasing attention over the years. Recently, Gammage et al. (2014) reported progress in their findings on the use of ZFN to target and cleave predetermined loci in mtDNA (Gammage et al., 2014). They generated a mitochondrially targeted ZFN carrying two cleavage domains bound to the same protein that selectively eliminates the deleterious mtDNA, as well as a mtDNA region most frequently associated with diseases, which contains several transfer RNAs and structural genes of the OXPHOS apparatus (Gammage et al., 2014). Meanwhile, mito-TALENs are used to cleave specific sequences in mtDNA with the goal of eliminating mitochondria carrying pathogenic point mutations. Additionally, in a recent study, this strategy was reported to target two clinically important mtDNA point mutations associated with mitochondrial diseases, including myoclonus epilepsy with ragged red fibers (MERRF) and MELAS/Leigh syndrome (Hashimoto et al., 2015). As a result, the mutation load was successfully reduced *in vitro*, as shown by decreased biochemical OXPHOS defects. However, the location of the mutations and the size of the mito-TALEN remain the major challenges in translating the mito-TALEN approach into a clinical setting (Hashimoto et al., 2015). Another study showed that a mito-TALEN specific for the mtDNA region harboring the m.5024C>T mutation was cloned into an adeno-associated virus and phage vector to test its role in regulating mtDNA heteroplasmy *in vivo* in a mouse model (Lehmann et al., 2015, Bacman et al., 2018). A significant decrease in the mutant/wild-type ratio expressed in skeletal and cardiac muscle compared to nontargeted tissue was observed after the systemic delivery of AAV9–mito-TALEN (Bacman et al., 2018).

The nontargeted vectors for the delivery of nucleases can be briefly categorized as adenoviruses, adeno-associated viruses, and nonviral vectors. These vectors are vital media for the realization of MGE *in vivo* since they are always applied in combination with mitochondria-targeted nucleases or endonucleases for delivery and effective functioning in cells. Studies have shown that, in some cases, mitochondrial genes can be integrated into the nuclear genome by the use of nontargeted viral vectors, a process mimicking the evolutionary nuclear movement of mitochondrial genes.

Recombinant adenovirus type 5 (rAd5) was used *in vivo* in NZB/BALB mice to deliver mito-ApaLI to the mitochondria. rAd5-expressing mitoREs were successful in eliminating BALB mtDNA in the heart (Bacman et al., 2010). However, due to the short expression window and promotion of a strong immunological response, rAd5 is not popularly used for *in vivo* applications (Wold and Toth, 2013).

AAV is the most commonly used viral vector for gene therapy due to its long-term expression of the transgene and history of well-established clinical safety (Pankajakshan et al., 2012). Different serotypes of AAV vectors can be generated to transduce a wide range of tissues and cells (Zincarelli et al., 2008; Ellis et al., 2013). The episomal expression feature of AAV makes it long-lasting in tissues, and the low frequency of integration adds to its safety features. It has also been demonstrated in Leber’s hereditary optic neuropathy, a mitochondrial disorder resulting in acute vision loss in young adulthood caused by mutations in NADH ubiquinone oxidoreductase subunits (including ND4) through the use of AAV (Roth and Marson, 2021). The nuclear insertion of corrected copies of the causative ND4 gene resulted in vision improvements in some human patients (Zhang et al., 2017), and the viral injection of mitochondrial targeting sequence–modified AAVs containing corrected copies of ND4 into the eyes of affected mice similarly resulted in vision improvements (Yu et al., 2012). However, the disadvantages of AAV include a packaging size limited to less than 5 kb, which restricts the size of the transgene that can be carried and delivered (Naso et al., 2017). Nevertheless, there have been records of immune response in patients exposed to AAV, requiring great caution in its clinical application (Naso et al., 2017; Vandamme et al., 2017). Although there are many AAV serotypes available for use, many have broad tropisms that may transduce nontarget tissues. Moreover, alteration of AAV capsid proteins allows selective and precise tissue transduction and deceases off-target effects (Wang et al., 2018; Zekonyte et al., 2020).

Third, nonviral vectors circumvent the major problem of immune effects induced by the viral vectors. Nanoparticles for chemical nonviral nucleic acid delivery usually come in the form of lipoplexes (DNA/catatonic lipids), polyplexes (DNA/catatonic polymers), and lipopolyplexes (DNA/catatonic polymers/catatonic lipids) for transferring large plasmid DNA molecules and small DNA molecules in the form of oligodeoxynucleotides and RNAs (Zekonyte et al., 2020). Inorganic nanoparticles can be made of silica or gold, and organic particles can be made of lipid emulsions, lipid nanoparticles, and a wide variety of organic polymers (Zekonyte et al., 2020). Delivery of these molecules includes physical methods, such as direct injection, electroporation, sonoporation, and magnetofection, to induce gene delivery (Ramamoorth and Narvekar, 2015; Hardee et al., 2017). To date, the application of nanoparticles for MGE awaits further exploration.

### Opportunities and Challenges of MGE

To date, challenges associated with the delivery of gRNA into the mitochondria have been the major barrier to applying CRISPR/Cas editing technology to mtDNA (Prole et al., 2020). Notably, it was predicted that the combination of modified gRNA with mitochondrial targeting sequence-mediated shuttling of CRISPR/Cas9 proteins may eventually allow precise editing of the mitochondrial genome (Dan et al., 2021). As demonstrated in a 2010 study, polynucleotide phosphorylase (PNPASE) regulates the import of nuclear-encoded RNAs into the mitochondrial matrix *via* a 20-nucleotide stem-loop mitochondrial targeting signal (RP loop), which enables the relocation of nonmitochondrial transcripts into mitochondria in a PNPASE-dependent manner (Wang et al., 2010). Practically, this RP signal has been used to import wild-type tRNAs efficiently into human mitochondria (Wang et al., 2012). A recent study showed that the mtDNA could be targeted with engineered CRISPR/Cas9 to assess the mutagenesis outcome of nuclease-generated DSBs in human cells (Wang et al., 2021). Site-specific DSBs were generated *via Staphylococcus aureus* Cas9 fused with the mitochondrial targeting sequence of COX8A in mtDNA, providing novel potential for the treatment of mitochondrial diseases with MGE (Wang et al., 2021). Moreover, appending different signal sequences to gRNAs has recently been discussed, providing the first evidence that this is indeed a viable strategy to develop mito-CRISPRs (Anton et al., 2020). Therefore, it is still more than anticipated to witness further progress in the utilization of mtDNA editing technology in human infertility therapies.

Additionally, the generation of animal models is an important and indispensable approach for the exploration of the mitochondrial genome. To date, mouse models of mtDNA disease have either been generated by introducing pathogenic mtDNA mutations within mitochondria into mouse embryos and importing them into embryo stem (ES) cells that are implanted into embryos or by the generation and selection of mtDNA mutations produced by mtDNA mutator mice (Stewart, 2020). Novel MGE technologies offer the possibility of a more precise and efficient strategy to create animal models with certain mtDNA mutations and further give hope for the treatment of mitochondrial diseases. As has been proven, targeting functional nucleases into animal mitochondria, such as restriction enzymes, ZFNs, TALENs, or CRISPR, were realizable (Gammage et al., 2018b; Anton et al., 2020), but with debatable efficacy. However, thanks to the breakthrough of DdCBE, an optimal editing efficiency of up to 40 to 49% for the targeted base in human cell lines has been proven (Mok et al., 2020). Similar strategies have been employed to induce the ZFN-directed methylation of mtDNA (Minczuk et al., 2006) and to direct the paired FokI domains with mitochondrially targeted TALE or ZFN nucleases to limit nuclease activity to specific mtDNA target sequences (Bacman et al., 2018, Gammage et al., 2018b).

**Figure 2:**
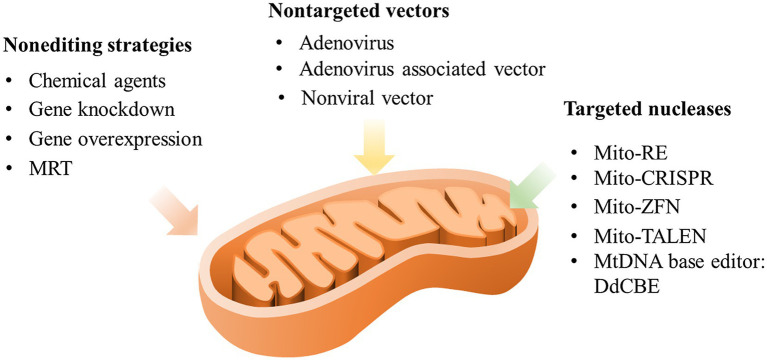
Possible manipulation strategies targeting mtDNA. MRT, mitochondrial replacement therapy; mito-RE, mitochondria-targeted restriction endonucleases; mito-CRISPR, mitochondria-targeted clustered regularly interspaced short palindromic repeats; mito-ZFN, mitochondria-targeted zinc finger nucleases; and mito-TALEN, mitochondria-targeted transcription activator-like effector nuclease.

Moreover, with the intracellular creation of mtDNA mutations becoming available, combined application with other model developing systems on the study infertility disease will be worth examining. It has been suggested that iPSCs or organoid systems could be combined with gene editing technology, eliminating the necessity of obtaining cells from patients bearing these rare diseases and allowing mutant and control cells or organoids to be studied under identical nuclear genetic backgrounds (Hatakeyama and Goto, 2016; Sison et al., 2018). In addition, targeted gene editing of mtDNA can provide new insights for understanding the novel functions of mtDNA and for developing new therapies for human maternally heritable mitochondrial infertility or other infertility diseases. However, it cannot be ignored that mtDNA editing is still only a promising option, and further studies and investigations are still required prior to implementing it in different cell types, organelles, and diseases as a new clinical therapy. Figure 2 summarizes the possible manipulation strategies for targeting mtDNA.

Although due to the ethical reasons, current studies on the human embryo are rather limited, we still believe that the development as well as the amelioration of the above-mentioned MGE technology will lead to the improvement of oocyte quality and the better prevention or treatment of certain mitochondrial improcreance and finally bring about the overall enhancement of the human fertility.

## Conclusion

Mitochondria not only are responsible for ATP production but also are related to a wide range of essential biological processes, including biosynthesis, calcium storage, cellular signaling, and immune response. They are also the only semiautonomous organelles with their unique mitochondrial genome that exerts a joint effect with the nuclear genome to manipulate organelle function. Unlike nDNA, circular mtDNA is tightly arranged and highly condensed, with a relatively high rate of mutation. The inheritance of mtDNA is strictly maternal, leading to a special “bottleneck” effect that tends to “purify” cell heteroplasmy into homoplasmy.

Since mitochondria play a vital role in the process of gametogenesis and fertilization, mitochondrial dysfunction and deleterious mtDNA have been shown to induce a pernicious impact on sperm or oocyte quality and finally lead to infertility. With the rapid development of gene editing technology, MGE has gained increasing attention and made compelling breakthroughs; consequently, targeting mtDNA opens a new door for the treatment of mito-related infertility. However, due to barriers to accessibility, efficacy, controllability, and safety, the maturation of this field and the clinical practice of this strategy still have a long way to go.

## Author Contributions

LF prepared the image and wrote the manuscript. YX-L, YL, and H-zL joined the discussion for the revision. H-zL and YY revised the manuscript. LF and YY designed the review article. All authors contributed to the article and approved the submitted version.

### Conflict of Interest

The authors declare that the research was conducted in the absence of any commercial or financial relationships that could be construed as a potential conflict of interest.
